# The T-box transcription factor Brachyury regulates epithelial–mesenchymal transition in association with cancer stem-like cells in adenoid cystic carcinoma cells

**DOI:** 10.1186/1471-2407-12-377

**Published:** 2012-08-29

**Authors:** Miyuki Shimoda, Tsuyoshi Sugiura, Ikumi Imajyo, Kotaro Ishii, Satomi Chigita, Katsuhiro Seki, Yousuke Kobayashi, Kanemitsu Shirasuna

**Affiliations:** 1 Division of Maxillofacial Diagnostic and Surgical Sciences, Department of Oral and Maxillofacial Surgery, Graduate School of Dental Science, Kyushu University, 3-1-1 Maidashi, Higashi-ku, Fukuoka, 812-8582, Japan

**Keywords:** Brachyury, Epithelial mesenchymal transition (EMT), Cancer stem cell (CSC), Adenoid cystic carcinoma (AdCC)

## Abstract

**Background:**

The high frequencies of recurrence and distant metastasis of adenoid cystic carcinoma (AdCC) emphasize the need to better understand the biological factors associated with these outcomes. To analyze the mechanisms of AdCC metastasis, we established the green fluorescence protein (GFP)-transfected subline ACCS-GFP from the AdCC parental cell line and the metastatic ACCS-M GFP line from an *in vivo* metastasis model.

**Methods:**

Using these cell lines, we investigated the involvement of the epithelial–mesenchymal transition (EMT) and cancer stem cell (CSCs) in AdCC metastasis by real-time RT-PCR for EMT related genes and stem cell markers. Characteristics of CSCs were also analyzed by sphere-forming ability and tumorigenicity. Short hairpin RNA (shRNA) silencing of target gene was also performed.

**Results:**

ACCS-M GFP demonstrated characteristics of EMT and additionally displayed sphere-forming ability and high expression of EMT-related genes (Snail, Twist1, Twist2, Slug, zinc finger E-box binding homeobox 1 and 2 [Zeb1 and Zeb2], glycogen synthase kinase 3 beta [Gsk3β and transforming growth factor beta 2 [Tgf-β2]), stem cell markers (Nodal, Lefty, Oct-4, Pax6, Rex1, and Nanog), and differentiation markers (sex determining region Y [Sox2], Brachyury, and alpha fetoprotein [Afp]). These observations suggest that ACCS-M GFP shows the characteristics of CSCs and CSCs may be involved in the EMT of AdCC. Surprisingly, shRNA silencing of the T-box transcription factor Brachyury (also a differentiation marker) resulted in downregulation of the EMT and stem cell markers. In addition, sphere-forming ability, EMT characteristics, and tumorigenicity were simultaneously lost. Brachyury expression in clinical samples of AdCC was extremely high and closely related to EMT. This finding suggests that regulation of EMT by Brachyury in clinical AdCC may parallel that observed *in vitro* in this study.

**Conclusions:**

The use of a single cell line is a limitation of this study. However, parallel data from *in vitro* and clinical samples suggest the possibility that EMT is directly linked to CSCs and that Brachyury is a regulator of EMT and CSCs.

## Background

Adenoid cystic carcinoma (AdCC) is one of the most common malignant tumors of the salivary glands and is characterized by unique clinical features and behavior. AdCC grows slowly but spreads relentlessly into adjacent tissues. The frequencies of recurrence and distant metastasis of AdCC are very high, with 40–60% of AdCC patients developing distant metastases to the lungs, bone, and soft tissues [1,2]. Therefore, distant failure remains a significant obstacle to the long-term cure of patients with AdCC, emphasizing the need to better understand the biological factors associated with AdCC distant metastases.

To identify the factors that mediate AdCC metastasis, we established 3 AdCC cell lines expressing green fluorescent protein (GFP) from the ACCS cell line by using orthotopic transplantation and *in vivo* selection in the nude mouse: the parental ACCS-GFP, the highly tumorigenic ACCS-T GFP, and the metastatic ACCS-M GFP. These cells were subjected to DNA microarray analysis, and the results revealed significantly altered biological processes in ACC-M GFP, including events related to cell adhesion and signaling. In particular, a significant downregulation of cell adhesion molecules such as E-cadherin and integrin subunits was observed. We confirmed the loss of E-cadherin and integrins and gain of vimentin in ACCS-M GFP, suggesting that the epithelial–mesenchymal transition (EMT) is a putative event in AdCC metastasis and induces tumor cell dissemination from the primary tumor site [3].

Recent evidence has demonstrated that the EMT is involved in a dedifferentiation program in epithelial tumor progression. This process interrupts cell-to-cell contact in a homocellular fashion in tumors and allows the dissemination of a single cell from the primary site. Therefore, EMT may be one of the important phenotypic alterations promoting nonmetastatic tumor transition to metastatic carcinoma [4,5].

The EMT program triggered during tumor progression appears to be controlled by genes normally expressed in the early embryo, including Twist, Snail, Slug, Goosecoid, and Sip1 [6-11]. The transcription factors encoded by these genes can impart the traits of mesenchymal cells to tumor cells, including motility and invasiveness. The expression of Twist, for example, is elevated in various types of cancers including breast, prostate, gastric, and melanoma [12]. In addition, the T-box transcription factor Brachyury, a gene required for mesoderm formation during the development process [13-15], is also reportedly able to promote the EMT in human carcinoma cell lines [16]. The latter study additionally revealed that overexpression of Brachyury (also described as a mesoderm differentiation marker) in human carcinoma cells induced changes characteristic of EMT. Therefore, mechanisms similar to EMT in human developmental processes are proposed to control EMT in cancer cells.

Independent of these studies, neoplastic tissue studies have provided evidence of self-renewing, stem-like cells within tumors, termed cancer stem cells (CSCs). CSCs constitute a minority of neoplastic cells within a tumor and are defined operationally by their ability to seed new tumors. For this reason, they have also been called “tumor-initiating cells” [17]. During the process of tumor metastasis, which is often enabled by EMT [18], disseminated cancer cells presumably require a self-renewal capability similar to that exhibited by stem cells in order to spawn macroscopic metastases. This phenomenon raises the possibility that the EMT process, which enables cancer cell dissemination, may also impart a self-renewal capability to disseminating cancer cells. Indeed, emerging evidence of a direct interaction between EMT and CSCs has been recently reported [16,19-22]. CSCs were shown to be resistant to chemotherapy and radiotherapy [21,23] and these studies therefore provide a new concept for therapies that target CSCs [24-28].

Given these reports and our previous results, we hypothesized that the EMT in our AdCC metastasis model involves AdCC stem cells and that the development of anti-CSC therapy may be effective in the treatment of AdCC. In this study, we demonstrate evidence of a direct interaction between the EMT and CSCs in the highly metastatic AdCC subclone ACCS-M GFP. We also report that the T-box transcription factor Brachyury [29-31] is a possible central regulator of CSCs and the EMT in AdCC cells.

## Results

### AdCC cells with EMT characteristics also have CSC-like phenotypes

**Figure 1:**
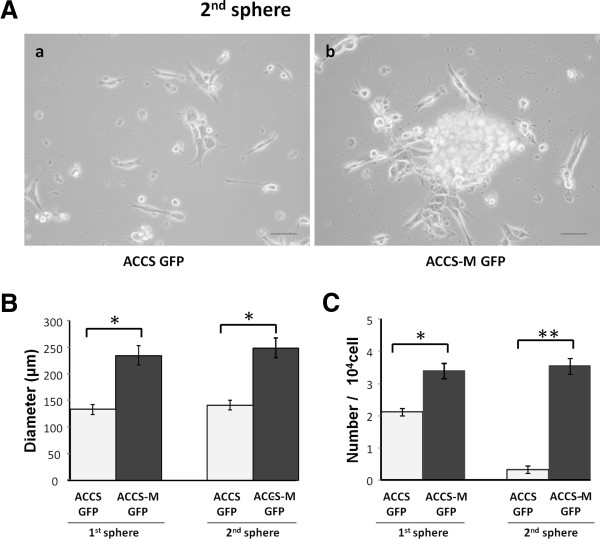
**Cells with EMT alterations show sphere-forming ability.** ACCS-GFP and ACCS-M GFP cells were cultured at a density of 5 × 10^4^ cells/mL in serum-free medium containing 40 ng/mL bFGF and 20 ng/mL EGF for floating culture for 10 days (primary spheres). For secondary spheres, primary spheres (day 10) were dissociated into single cells and further cultured at a density of 1 × 10^4^ cells/mL for 10 days. Spheres were observed under a phase contrast microscope (**A**). Sphere diameters were measured (**B**), and spheres with a diameter >100 μm were counted. Sphere numbers were standardized as sphere number/10^4^ cells originally cultured (**C**) in each sphere period. Experiments were performed in triplicate, and the values were averaged. Bars indicate the standard deviation. Data significance was analyzed by Student’s *t*-test. *P < 0.05, **P < 0.01.

We previously isolated the highly metastatic and tumorigenic AdCC subline ACCS-M GFP from nonmetastatic (0% incidence) and low tumorigenic (22.2% incidence) parental ACCS GFP cells using *in vivo* selection as described in the Methods [3]. ACCS-M GFP exhibited high tumorigenicity (100% incidence), high frequency of spontaneous metastasis to submandibular lymph nodes (100% incidence), and significant characteristic changes of the EMT, such as loss of E-cadherin and gain of vimentin [3]. Ample evidence has accumulated indicating that the EMT is closely correlated with CSCs. AdCC cells with the EMT phenotype (ACCS-M GFP) also showed significant tumorigenicity, which is an important phenotype of CSCs [3]. Therefore, we assessed the stemness of ACCS cell lines with the sphere-forming assay. The parental ACCS GFP cells demonstrated weak sphere-forming capacity in diameter and number, whereas ACCS-M GFP cells showed significant sphere-forming capacity (Figure 1). The sphere diameter of ACCS-M GFP was approximately twice the diameter of ACCS GFP in the primary and secondary spheres (Figure 1B). Furthermore, the number of spheres was more significantly different in the secondary spheres than in the primary spheres. The number of spheres of ACCS-M GFP was approximately 10 times higher than that of ACCS GFP (Figure 1C). These data suggest that ACCS-M GFP cells have self-renewal (sphere-forming) ability.

### AdCC cells with EMT characteristics express EMT-related genes and stem-cell markers

**Figure 2:**
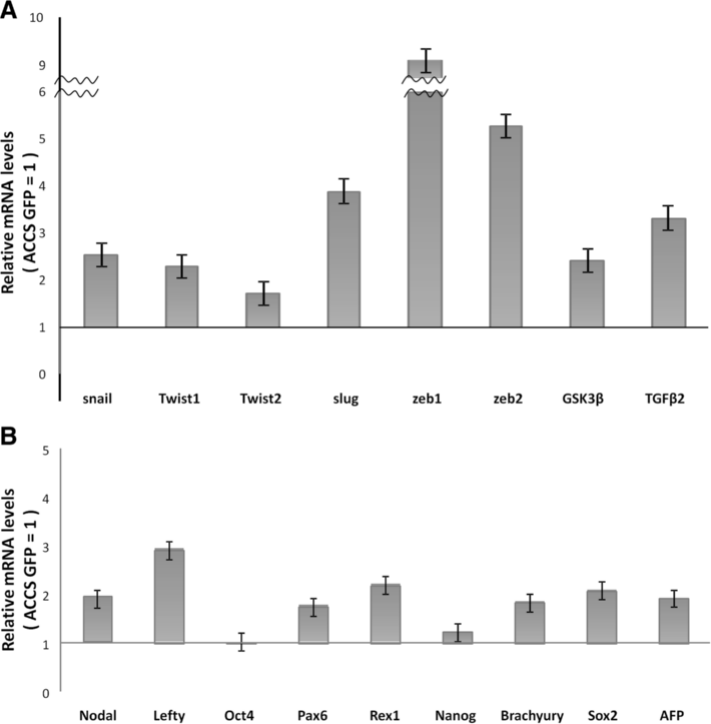
**Analysis of gene expression related to EMT and CSCs by real-time PCR.** The mRNA expression levels of the indicated EMT-related genes (**A**) and embryonic stem cell markers (Nodal, Pax6, Rex1, Lefty, Oct-4, and Nanog) and differentiation markers (mesoderm marker: Brachyury; ectoderm marker: Sox2; endoderm marker: AFP) (**B**) in ACC cells were quantified by real-time RT-PCR. Each mRNA level was compared between ACCS GFP and ACCS-M GFP, and data are shown as relative mRNA levels to β-actin mRNA. Experiments were performed in triplicate, and the number of adhered cells was averaged. Bars indicate the standard deviation.

We next quantified the expression levels of possible CSC markers by real-time RT-PCR, which are shown as relative mRNA levels compared to β-actin mRNA (Table 1). ACCS cells expressed higher levels of genes such as Snail, Slug, Tgf-β2, Pax6, and Brachyury than other genes tested. Expression levels of EMT-related genes such as Snail, Twist1, Twist2, Slug, zinc finger E-box binding homeobox 1 and 2 (Zeb1 and Zeb2), glycogen synthase kinase 3 beta (Gsk3β and transforming growth factor beta 2 (Tgf-β2) were elevated from 2-fold to 9-fold in ACCS-M GFP compared to ACCS GFP (Figure 2A). This increased expression in ACCS-M GFP was especially apparent with Slug (approximately 4-fold), Zeb1 (approximately 9-fold), and Zeb2 (approximately 5.5-fold). Stem cell markers (Nodal, Lefty, Oct-4, Pax6, Rex1, and Nanog) and differentiation markers (sex determining region Y [Sox2], Brachyury, and Afp) were also overexpressed in ACCS-M GFP, with the exception Oct-4 and Nanog (Figure 2B). Together, these data suggest that ACCS-M GFP cells have CSC-like phenotypes and are related to the EMT.

**Table 1:** Analysis of gene expression levels related to EMT and CSCs by real-time PCR

**Genes**	**Cell line**	**Relative mRNA level (normalized to β-actin)**	
		**Mean expression levels**	**SD**
Snail	ACCS	2.02 × 10^-2^	4.18 × 10^-3^
	ACCSM	5.14 × 10^-2^	2.62 × 10^-3^
Slug	ACCS	1.05 × 10^-2^	4.82 × 10^-3^
	ACCSM	4.14 × 10^-2^	2.92 × 10^-3^
Twist1	ACCS	1.47 × 10^-4^	5.24 × 10^-5^
	ACCSM	3.37 × 10^-4^	1.12 × 10^-5^
Twist2	ACCS	4.68 × 10^-3^	2.67 × 10^-4^
	ACCSM	8.04 × 10^-3^	1.38 × 10^-4^
Zeb1	ACCS	3.21 × 10^-3^	5.13 × 10^-4^
	ACCSM	2.92 × 10^-2^	6.16 × 10^-4^
Zeb2	ACCS	1.40 × 10^-3^	6.23 × 10^-4^
	ACCSM	7.39 × 10^-3^	4.12 × 10^-4^
Tgf-β2	ACCS	5.43 × 10^-2^	3.56 × 10^-2^
	ACCSM	1.81 × 10^-1^	7.52 × 10^-2^
Gsk3β	ACCS	3.39 × 10^-3^	1.82 × 10^-4^
	ACCSM	8.73 × 10^-3^	6.43 × 10^-4^
Nodal	ACCS	3.72 × 10^-3^	4.36 × 10^-4^
	ACCSM	7.14 × 10^-3^	2.15 × 10^-4^
Pax 6	ACCS	6.34 × 10^-2^	4.26 × 10^-2^
	ACCSM	1.10 × 10^-1^	8.21 × 10^-2^
Rex 1	ACCS	1.12 × 10^-3^	2.42 × 10^-4^
	ACCSM	2.45 × 10^-3^	2.93 × 10^-4^
Lefty	ACCS	3.39 × 10^-3^	1.51 × 10^-4^
	ACCSM	9.88 × 10^-3^	7.12 × 10^-4^
Brachyury	ACCS	1.55 × 10^-2^	5.32 × 10^-3^
	ACCSM	2.83 × 10^-2^	6.24 × 10^-3^
Sox 2	ACCS	9.35 × 10^-3^	2.12 × 10^-4^
	ACCSM	1.94 × 10^-2^	8.92 × 10^-4^
AFP	ACCS	9.50 × 10^-3^	3.52 × 10^-4^
	ACCSM	1.83 × 10^-2^	9.96 × 10^-4^

### Knockdown of the T-box transcription factor Brachyury downregulates EMT-related genes and stem-cell markers

**Figure 3:**
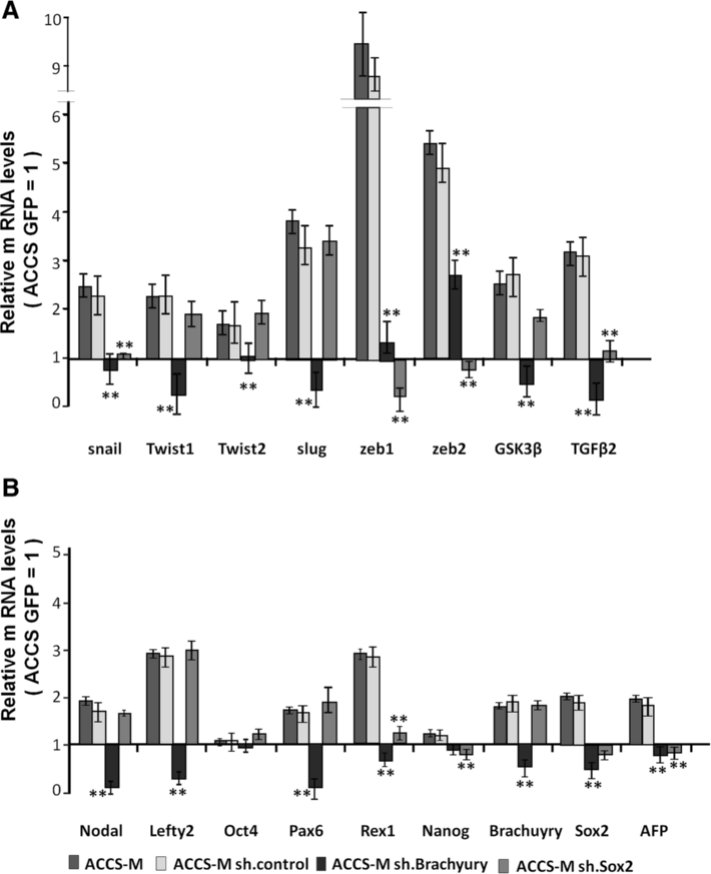
**Effect of Brachyury shRNA on ACCS-M GFP gene expression related to EMT and CSCs.** ACCS-M-sh. control was generated by the transfection of ACCS-M GFP cells with control vector. ACCS-M sh Brachyury and ACCS-M sh Sox2 were generated by the transfection of ACCS-M GFP cells with Brachyury shRNA and Sox2 shRNA, respectively. The mRNA expression levels of the indicated genes in ACCS-M GFP cells and derivatives were quantified by real-time RT-PCR. Each mRNA level was compared with ACCS GFP, and data are shown as relative mRNA levels (ACCS GFP = 1). The expression levels of EMT-related genes (**A**) and stem cell markers and differentiation markers (**B**) are shown. Experiments were performed in triplicate, and the number of adhered cells was averaged. Bars indicate the standard deviation.

We next sought direct evidence of linkage between EMT and CSCs with the aim to simultaneously reveal the central regulator(s) of CSC stemness. Several of the CSC markers in Figure 2 are transcription factors, and recent reports have demonstrated that the T-box transcription factor Brachyury promotes the EMT in human tumor cells [16,32]. Therefore, we focused on the possibility that Brachyury regulates not only EMT but also CSC stemness. We also focused on SOX2, which has also been reported as one of the key element genes for embryonic or pluripotent stem cells. We used a stable transfection system for Brachyury and SOX2 short hairpin RNA (shRNA) in lentiviral plasmids. Following Brachyury and SOX2 knockdown, the expression levels of all examined CSC markers were assessed by real-time RT-PCR (Figure 3). Each mRNA level was compared with ACCS GFP, and data are shown as relative mRNA levels (ACCS GFP = 1). The expression levels of EMT-related genes (Figure 3A) and stem cell markers and differentiation markers (Figure 3B) are shown. The mRNA levels of all CSC markers decreased in Brachyury-knockdown ACCS-M GFP cells (ACCS-M shBra) compared to ACCS GFP. In contrast, SOX2-knockdown ACCS-M GFP cells (ACCS-M shSOX2) demonstrated specific downregulation of only *Snail*, *Zeb1*, *Zeb2*, *Tgfβ2*, *Rex1*, *Nanog*, and Afp mRNA. Importantly, SOX2 knockdown failed to regulate *Brachyury* mRNA expression. These results strongly suggest that Brachyury is a central regulator of CSC and EMT.

### Knockdown of the T-box transcription factor Brachyury negates EMT phenotypes

We then confirmed the EMT phenotype in ACCS-M shBra and ACCS-M shSOX2. The protein level of β-catenin was increased and shifted to higher molecular weight in ACCS-M GFP. The protein level of β-catenin was decreased in ACCS-M shBra and ACCS-M shSOX2 cells, reaching similar levels to that observed in ACCS GFP; however, the molecular weight of β-catenin was increased, similar to ACCS-M GFP.

**Figure 4:**
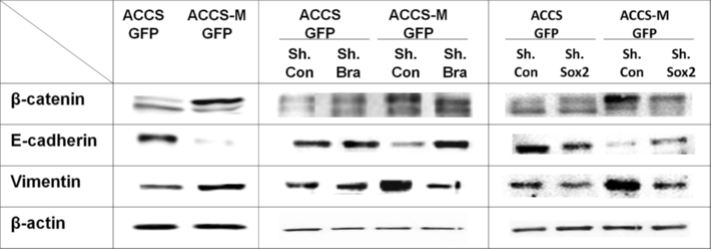
**Silencing of Brachyury recovers the epithelial signature of ACCS-M GFP cells.** sh control (sh. con) cells were generated by the transfection of ACCS GFP or ACCS-M GFP cells with control vector. sh Brachyury (sh.Bra) or sh SOX2 (sh.SOX2) cells were generated by the transfection of ACCS GFP or ACCS-M GFP cells with Brachyury shRNA or SOX2 shRNA. Cells were cultured for 24 h on culture dishes, and cell lysates were prepared and resolved using 10% SDS-PAGE. The levels of EMT-related biomarkers were detected by immunoblotting with antibodies against the indicated proteins. All experiments were performed at least in triplicate, and representative results are shown.

The protein level of E-cadherin was increased in ACCS-M shBra and recovered to the approximate level observed in ACCS GFP cells, but recovery in ACCS-M shSOX2 was incomplete (Figure 4). Vimentin protein level was decreased in both ACCS-M shBra and ACCS-M shSOX2 cells compared to ACCS-M GFP, reaching similar levels to that observed in ACCS GFP.

### Knockdown of the T-box transcription factor Brachyury inhibits sphere-forming capacity

**Figure 5:**
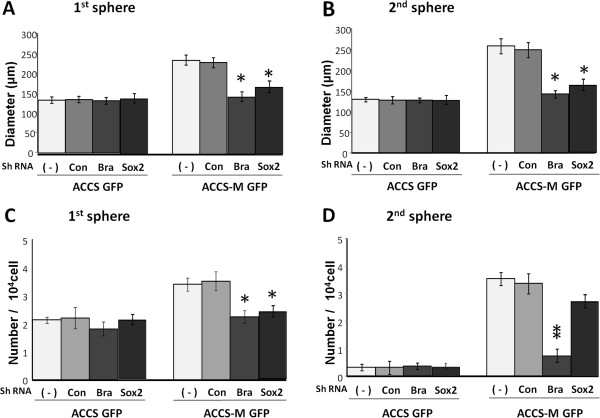
**Brachyury silencing reduces the sphere-forming ability of ACCS-M GFP cells.** ACCS cells and ACCS derivatives transfected with shRNAs (−, untreated control; con, control shRNA; Bra, Brachyury shRNA; Sox2, SOX2 shRNA) were cultured, and sphere-forming ability was quantified as described in the legend for Figure 1. Sphere diameters were measured (**A**), and spheres with a diameter >100 μm were counted. Sphere numbers were standardized as sphere number/10^4^ cells originally cultured (**B**) in each sphere period. Experiments were performed in triplicate, and the values were averaged. Bars indicate the standard deviation. Data significance was analyzed by Student’s *t*-test. *P < 0.05, **P < 0.01.

We examined the self-renewal capability of ACCS-M shBra and ACCS-M shSOX2 by sphere-forming assay. Similar to ACCS GFP cells, ACCS-M shBra and ACCS-M shSOX2 lost sphere-forming capacity with respect to the diameter of the primary (Figure 5A) and secondary spheres (Figure 5B) and with respect to the number of cells in the primary spheres (Figure 5C). Furthermore, the number of spheres was more significantly lower in the secondary spheres than in the primary spheres, and ACCS-M shBra significantly reduced sphere number in comparison to ACCS-M shSOX2 (Figure 5D). These data suggest that Brachyury is a more important regulator of EMT and CSC than SOX2.

### Knockdown of the T-box transcription factor Brachyury inhibits tumorigenicity and metastasis *in vivo*

**Figure 6:**
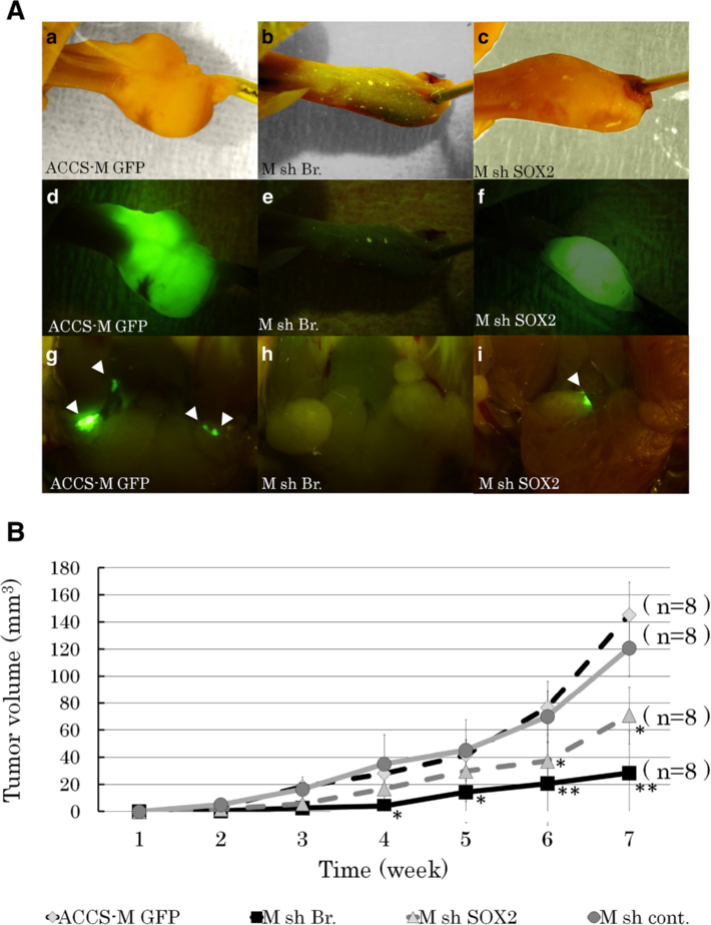
**Brachyury silencing reduces the tumorigenicity and metastasis of ACCS-M GFP cells.A**. ACCS-M GFP derivatives transfected with shRNAs (untreated control, ACCS-M GFP [**a**, **d**, **g**]; Brachyury shRNA, M sh.Bra [**b**, **e**, **h**]; SOX2 shRNA, M sh.SOX2 [**c**, **f**, **i**]) were injected into the tongues of nude mice and examined to detect tumors in tongues (a–f) and spontaneous metastases in submandibular lymph nodes (**g**–**i**). Observations with the naked eye (**a**–**c**) and the excitation of GFP (**d**–**i**) are shown. Sites of lymph node metastasis are labeled with arrowheads. Note that GFP enables the detection of micro-metastasis in the lymph nodes. **B**. The primary tumor volumes were measured weekly, calculated as the length × width × thickness, and mice were sacrificed when the primary tumor volume reached 100 mm^3^. Tumor growth curves for ACCS derivatives ACCS-M GFP (diamond), sh.Bra (square), sh.SOX2 (triangle), and sh cont. (control shRNA, circle) are shown. Experiments were performed in 6 mice for each ACCS derivative, and the values were averaged. Bars indicate the standard deviation. Data significances between ACCS-M GFP and other ACCS derivatives were analyzed by Student’s *t*-test. *P < 0.05, **P < 0.01.

The effect of Brachyury knockdown on ACCS-M GFP tumorigenicity and metastasis *in vivo* was examined using a mouse metastasis model established and reported by Ishii *et al.*[3]*.* Figure 6A shows a typical tumor in tongue (a–c), its GFP excitation (d–f), and submandibular lymph node metastasis (g–i). Remarkably, ACCS-M shBra sometimes failed to develop into tongue tumor (50% tumorigenicity), and metastasis was completely inhibited. ACCS-M shSOX2 also reduced tumorigenicity (87.5%) and metastasis (87.5%), but the impact of inhibition was more relevant with ACCS-M shBra (Table 2). Tumor growth rate was also significantly inhibited in ACCS-M shBra cells (Figure 6B).

**Table 2:** Tumorigenicity and metastasis of each cell line

	**Tumorigenicity**		**Metastasis**	
** Cell line**	**Tumorigenic mouse (%)**	**Tumor volume (mm**^**3**^**)**	^*^SMLN (%)	**LUNG (%)**
ACCS-M GFP	8/8 (100)	145.3	8/8 (100)	7/8 (87.5 )
ACCS-M sh cont.	8/8 (100)	121.4	8/8 (100)	7/8 (87.5%)
ACCS-M sh Br.	4/8 (50)	28.3	N.D	N.D
ACCS-M sh SOX2	7/8 (87.5)	37.2	7/8 (87.5)	3/8 (37.5%)

N.D: not detected

^*^Submandibular lymph nodes.

### Expression and molecular localization of Brachyury and EMT markers in oral AdCC lesions

**Figure 7:**
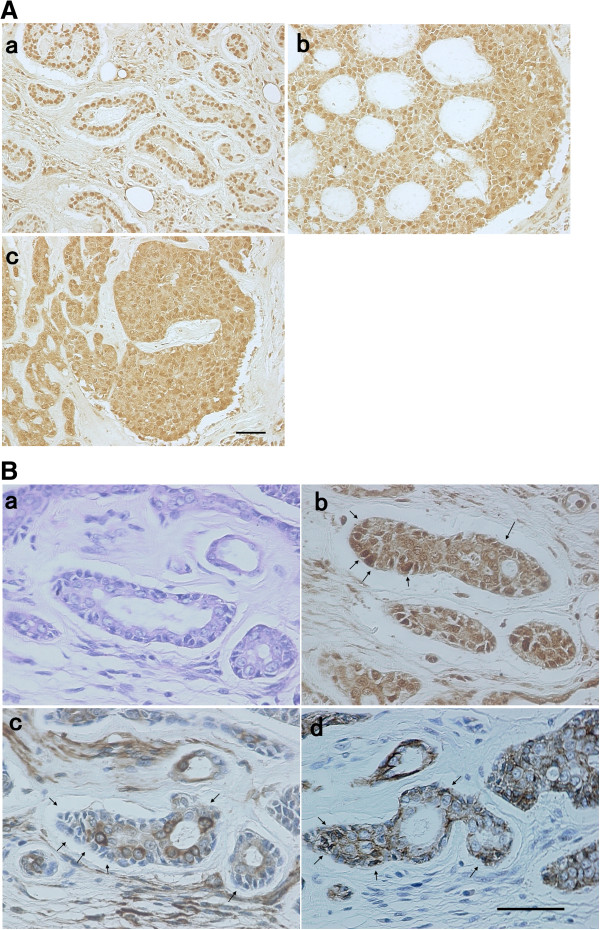
**Immunohistochemical analysis of Brachyury, E-cadherin, and vimentin in AdCC tissue.** Sections of 4-μm thickness were used for the histopathological and immunohistochemical analysis as described in the Methods. **A**. Representative staining pattern of Brachyury on AdCC (**a**: tubular pattern, **b**: cribriform pattern, **c**: solid pattern). Bar = 50 μm**.B**. Serial AdCC sections were stained with HE (**a**) or immunostained with Brachyury (**b**), E-cadherin (**c**), and vimentin (**d**). Bar = 50 μm.

We examined the expression and expression pattern of Brachyury in oral AdCC lesions using immunohistochemistry. Figure 7A shows the representative staining pattern of Brachyury on AdCC (a: tubular pattern. b: cribriform pattern, c: solid pattern). Brachyury was localized to the cytoplasm and/or nucleus of AdCC cells. We examined 21 AdCC samples, and all samples demonstrated positive expression of Brachyury in AdCC cells (positive expression rate =100%, Table 3). To find evidence that Brachyury was associated with EMT, we analyzed localization of Brachyury (Figure. 7B-b), E-cadherin (Figure. 7B-c), and vimentin (Figure. 7B-d) in AdCC tissue by immunofluorescence staining of serial sections. The lateral layer of the AdCC cells expressed Brachyury in the nucleus (Figure. 7B-b, arrowheads). These cells lost expression of E-cadherin (Figure. 7B-c, arrowheads) and gained expression of vimentin (Figure. 7B-d, arrowheads).

**Table 3:** The clinical features and Brachyury expression patterns of patients with oral AdCC

**Pt.**	**Sex**	**Age (y)**	**Region**	**Pathological classification**	**Lymph node involvement**	**Distant metastasis**	**Brachyury expression**	
							**Nucleus**	**Cytoplasm**
1	M	56	Sublingual gland	Cribriform	-	-	+	+
2	F	83	Sublingual gland	Cribriform	-	-	+	+
3	F	71	Sublingual gland	Solid	-	Lung	+	+
4	M	70	Sublingual gland	Cribriform	-	-	+	+
5	M	34	Palate	Tubular	-	-	+	+
6	F	25	Palate	Cribriform	-	-	+	+
7	F	64	Upper gingiva	Tubular	-	Lung bone	+	+
8	F	58	Palate	Solid	+	-	+	+
9	F	76	Sublingual gland	Solid	+	-	+	+
10	F	75	Sublingual gland	Cribriform	+	-	+	-
11	M	58	Palate	Cribriform	+	-	+	+
12	F	80	Sublingual gland	Cribriform	-	-	+	+
13	M	61	Lower gingiva	Solid	-	Lung brain	+	+
14	F	57	Upper gingiva	Cribriform	-	Lung bone	+	+
15	M	63	Palate	Solid	+	Liver	+	+
16	F	59	Palate	Tubular	-	-	+	+
17	F	65	Sublingual gland	Cribriform	-	-	+	+
18	M	65	Upper gingiva	Solid	-	-	+	+
19	F	70	Sublingual gland	Solid	+	Lung	+	+
20	M	69	Palate	Cribriform	-	-	+	+
21	F	48	Upper gingiva	Cribriform	-	Lung	+	+

## Discussion

Cancer metastasis is the most crucial event directly influencing patient prognosis. Recent studies suggest that the EMT is strongly correlated with cancer invasion and metastasis [33,34]. In contrast, CSCs have gained attention as targets for cancer treatment because they show chemo- and radioresistance [21,35-37]. More recently, EMT was reported to promote the CSC signature [19,38-40]; however, the regulatory mechanism of CSC and EMT is still unclear.

We demonstrated a direct correlation between EMT and CSCs in AdCC cells. Importantly, the EMT we analyzed in this study was developed from an *in vivo* model and was not artificially isolated [33], exogenous [41,42], or genetically promoted [43], as described previously. Therefore, the findings that we report here strongly support the hypothesis that CSCs are involved in the EMT. This study is the first to identify Brachyury as a regulator for both EMT and CSC characteristics. This conclusion is based on the observation that Brachyury knockdown resulted in simultaneous loss of all stem cell markers and loss of EMT and CSC phenotypes in morphological and biochemical assays.

The classification of EMT into 3 subtypes based on the biological and biomarker context in which they occur has been proposed [44,45]. EMT associated with organ development is referred to as type 1 EMT, and EMT associated with wound healing and tissue regeneration are type 2 EMT. EMT in cancer progression and metastasis is categorized as type 3 EMT. Multiple extracellular signals including TGF-β, receptor tyrosine kinases, Notch, nuclear factor kappa B (NFκB), and Wnt can initiate the type 3 EMT program. The downstream intracellular signaling pathways and transcription factors that constitute this complex program demonstrate significant crosstalk, including multiple positive feedback loops [46,47].

This principle of EMT suggests that the phenomenon may be reversible if such extracellular signals are removed. However, our established cell line, ACCS-M GFP, is stable and does not change to a nonmetastatic phenotype after several passages. Recent data from mammary epithelial cells also demonstrate that continuous activation of the EMT leads to epigenetic alterations in cells that induce heritable effects to maintain the EMT state even after EMT-inducing signals or factors are no longer present [48]. Hence, under certain conditions such as *in vivo* selection, EMT can yield stable changes in phenotype and thus the lineage identity of cells. In these cells, all possible pathways initiating EMT are constitutively active without any stimulation, as shown in Figure 3. This characteristic may make the cells self-renewing, the most important phenotype of CSCs. This type of phenotypic alteration or cell selection is proposed to occur upon repeated chemotherapy or radiotherapy for cancer treatment *in vivo*.

Although much is known about the mechanisms or signals involved in type 1 and type 2 EMT [49,50], type 3 EMT-specific signaling still remains to be resolved in epithelial carcinoma cells. Our study indicates that one such possibility is the constitutive upregulation of TGF-β2 in ACCS-M GFP cells. TGF-β appears to be responsible for the induction or functional activation of a series of EMT-inducing transcription factors in cancer cells, notably Snail, Slug, ZEB1, Twist, Goosecoid, and FOXC2 [51-53]. Constitutive upregulation of TGF-β2 would therefore maintain the EMT or CSC status in an autocrine manner.

Brachyury is a T-box transcription factor with an evolutionarily conserved function in vertebrate development, whereby it is required for mesoderm formation [13-15]. Brachyury is also highly expressed in various human epithelial tumors and human tumor cell lines (lung, colon, and prostate carcinomas), but not in human normal adult tissues [32]. However, no studies have analyzed the role of Brachyury in tumor cells. Recently, Fernando *et al.*[16] reported that Brachyury promotes EMT in human carcinoma cell lines. Their study demonstrated that overexpression of Brachyury in human carcinoma cells induced EMT, including upregulation of mesenchymal markers, downregulation of epithelial markers, and increase in cell migration and invasion.

Downregulation of E-cadherin transcription is induced by Brachyury overexpression and partially mediated by Slug. In our model, Brachyury was overexpressed in the ACCS-M GFP (EMT cell line), and the expression level was 2-fold greater than that of the parental cell line. In contrast, overexpression of ZEB1 and ZEB2 in the EMT cell line was 5- and 9-fold higher, respectively, compared to parental cells. Surprisingly, Brachyury silencing by shRNA in ACCS-M GFP cells resulted in an almost complete inhibition of EMT-related genes and stem cell markers, including ZEB1 and ZEB2. This significant change induced by Brachyury silencing promoted the mesenchymal to epithelial transition (gain of E-cadherin and loss of vimentin) and loss of the CSC phenotype (sphere formation and tumorigenicity).

The mechanisms of Brachyury regulation of the EMT and stem cell-related genes are not certain. Brachyury and other members of the T-box transcription family preferentially bind to the palindromic consensus element AATTTCACACCTAGGTGTGAAATT, and a half-site (TCACACCT) of this consensus sequence is located at position −645 of the human E-cadherin promoter. Brachyury is able to bind to the E-cadherin promoter *in vitro*, although with low efficiency [16]. Other reports have suggested low-affinity binding of T-box proteins to a half consensus site, such as the one present in the E-cadherin promoter [54,55]. However, the *in vivo* binding of Brachyury to the half-site on the E-cadherin promoter could be greatly improved by interactions with accessory proteins or cofactors. Brachyury overexpression in tumor cells induces a concurrent enhancement of Slug expression, followed by the effective silencing of *E-cadherin* transcription as a result of Brachyury and Slug association within the E-cadherin promoter region [16].

The transcription factor Slug, but not Snail, has been shown to control desmosomal disruption during the initial and necessary steps of EMT in addition to repressing *E-cadherin* transcription [56,57]. Induction of EMT by FGF-1 treatment or Slug overexpression in the rat bladder carcinoma cell line NBT-II is also characterized by dissociation of desmosomes, with no change in E-cadherin expression [57]. Therefore, Slug may mainly control desmosomal proteins such as plakoglobin during the initial step of EMT and associate with Brachyury to regulate E-cadherin and accomplish EMT.

During the developmental process in vertebrates, Brachyury regulates downstream genes that are components of signaling pathways such as noncanonical Wnt/planar cell polarity (Wnt/PCP), NFκB, and TGF-β signaling [58]. Sox2 (SRY Sex Determining Region Y-Box2) is a member of the Sox (SRY-related HMG box) family of transcription factors. Sox2 regulates expression of multiple genes, especially stable expression of Oct-3/4, which is also a transcription factor that maintains stemness and pluripotency in normal stem cells. Recently, an association between SOX2 and EMT was also reported. Activation of SOX2 induces TGF-β downstream signaling including activation of Wnt, Notch, and Hedgehog signals, followed by induction of *Snail* mRNA expression to ultimately result in inhibition of *E-cadherin* transcription through induction of ZEB1/2 expression. This phenomenon is consistent with our mRNA expression results after SOX2 knockdown. Importantly, unlike Brachyury knockdown, SOX2 knockdown only inhibited genes downstream of TGF-β and failed to inhibit Brachyury expression. In contrast, Brachyury knockdown inhibited almost all the genes tested including *Sox2* and its downstream genes. Also of note, silencing of SOX2 inhibited EMT but not tumorigenicity and metastasis. Therefore, it is possible that Brachyury controls multiple functional signals related to EMT and CSC simultaneously. The impact of the simultaneous silencing effect of Brachyury on EMT and CSC phenotypes observed in this study support this hypothesis. Additionally, these data suggest the existence of a partial but direct link between the EMT and CSC and that Brachyury is one of the central regulators of EMT and CSC maintenance in AdCC cells.

The use of a single cell line is a limitation of this study. It is quite difficult to establish CSC-like cell lines *in vitro* and this is an obstacle to research in this field. However, parallel data from clinical samples support our hypothesis in part. Brachyury expression in clinical AdCC samples was extremely high (positive expression rate = 100%), and the data suggested a close relationship with EMT (loss of E-cadherin and gain of vimentin). Therefore, at least the regulation mechanism of EMT by Brachyury demonstrated in this study may also occur in clinical AdCC.

From a clinical perspective, CSC-targeted therapy should have strict selectivity for CSCs, which is a serious obstacle for most molecular targeted therapies presently used. Selective expression of Brachyury has been reported in various human tumors of epithelial origin, but not in most human normal adult tissues [32], a fact that strongly encourages the use of this molecule as a clinical therapeutic target.

## Conclusions

We conclude that the EMT is directly linked to CSC, and Brachyury is one of the central regulators of the EMT and CSC in our single cell line study. These results suggest that Brachyury is a potential therapeutic target for future anti-CSC treatments of AdCC.

## Methods

### Cells and culture

The human cell lines ACCS, ACCS GFP, and ACCS-M GFP were established in our laboratory as described previously [3]. In brief, the parental cell line ACCS and green fluorescence protein (GFP)-transfected subline ACCS-GFP displayed similar morphologies, growth rates, and tumorigenicity both *in vitro* and *in vivo*. Similar to the parental ACCS, the tumorigenicity of ACCS-GFP cells was low (22.2% incidence). Using ACCS-GFP cells, tumor formation in the tongues of nude mice injected with tumor cells was clearly observed under excitation light, while green fluorescence was not observed in the absence of tumors. We performed *in vivo* selection of clones with higher tumorigenicity by repeatedly recovering cells *in vitro* and transplanting them into the tongues of nude mice. Consequently, a subline exhibiting high tumorigenicity (100% incidence) and high frequency of spontaneous metastasis to submandibular lymph nodes (100% incidence), ACCS-M GFP, was obtained through this *in vivo* selection process. The histological and immunohistochemical features of ACCS-M GFP tumors were similar to the solid pattern of AdCC. The cell lines were maintained as a monolayer culture in Dulbecco's modified Eagle's medium (DMEM; Sigma-Aldrich, St. Louis, MO, USA) supplemented with 10% fetal bovine serum (ICN Biomedicals, Aurora, OH, USA), 2 mM l-glutamine, penicillin G, and streptomycin in a humidified incubator with an atmosphere of 5% CO_2_ at 37°C.

### Immunoblot analysis

To visualize cell adhesion molecules and their related proteins, cells were rinsed with phosphate-buffered saline (PBS) and lysed in ice-cold buffer (50 mM Tris–HCl [pH 7.5], 150 mM NaCl, 2 mM ethylene glycol tetraacetic acid [EGTA], and 1% Triton X-100) containing protease inhibitor cocktail (Sigma-Aldrich). The protein content of the lysates and fractionated samples was quantified using a protein assay kit (Bio-Rad Laboratories, Hercules, CA, USA). Equal amounts of protein from each sample were resuspended in sodium dodecyl sulfate (SDS) sample buffer (10% SDS, 62.5 mM Tris–HCl [pH 6.8], and 50% glycerol). Before electrophoresis, reduced samples were adjusted to 5% (v/v) 2-mercaptoethanol and boiled for 5 min. The samples were separated on 10% SDS–polyacrylamide gels and transferred electrophoretically onto nitrocellulose membranes (Bio-Rad Laboratories). After blocking with 5% skim milk in Tris-buffered saline containing 0.1% Tween-20, the membranes were incubated overnight with primary antibodies at 4°C, followed by horseradish peroxidase-conjugated secondary antibodies (DAKO, Carpinteria, CA, USA) for 1 h. The bound antibodies were visualized using ECL immunoblotting detection reagents (Amersham Pharmacia Biotech, Piscataway, NJ, USA). The following primary antibodies were used for immunoblotting: mouse monoclonal anti-vimentin (V9) purchased from Santa Cruz Biotechnology (Santa Cruz, CA, USA); mouse monoclonal anti-E-cadherin purchased from BD Transduction Laboratories (Franklin Lakes, NJ, USA); rabbit polyclonal anti-β-catenin purchased from Upstate (Temecula, CA, USA); and mouse monoclonal anti-β-actin (A5316) purchased from Sigma-Aldrich.

### Real-time RT-PCR

The mRNA expression levels of the indicated EMT-related genes, embryonic stem cell markers (Nodal, Pax6, Rex1, Lefty, Oct-4, and Nanog), and differentiation markers (mesoderm marker, Brachyury; ectoderm marker, Sox2; endoderm marker, AFP) in ACC cells were quantified by real-time RT-PCR.

Total RNA was extracted from ACCS cells using TRIzol (Invitrogen, Carlsbad, CA, USA) and used for first-strand cDNA synthesis. The mRNA levels were quantified in triplicate using a real-time PCR system with the Brilliant SYBR Green qPCR Kit (Stratagene, La Jolla, CA, USA). The specific primers for EMT, stem cells, and differentiation markers were as follows: hSnail (F) 5^′^-TCCACAAGCACCAAGAGTC-3^′^, (R) 5^′^-ATGGCAGTGAGAAGGATGTG-3^′^; hSlug (F) 5^′^-ACTGCTCCAAAACCTTCTCC-3^′^, (R) 5^′^-TGGTCAGCACAGGAGAAAATG-3^′^; hTwist1 (F)5^′^-CTCAGCTACGCCTTCTCG-3^′^, (R) 5^′^-ACTGTCCATTTTCTCCTTCTCTG-3^′^; hTwist2 (F) 5^′^-AGGAGCTCGAGAGGCAG-3^′^, (R) 5^′^-CGTTGAGCGACTGGGTG-3^′^; hZEB1 (F) 5^′^-CTCACACTCTGGGTCTTATTCTC-3^′^, (R) 5^′^-GTCTTCATCCTCTTCCCTTGTC-3^′^; hZEB2 (F) 5^′^-AAAGGAGAAAGTACCAGCGG-3^′^, (R) 5^′^-AGGAGTCGGAGTCTGTCATATC-3^′^; hTGF-β (F) 5^′^-TTAACATCTCCAACCCAGCG-3^′^, (R) 5^′^-TCCTGTCTTTATGGTGAAGCC-3^′^; hGSK3β (F) 5^′^-GGTCTATCTTAATCTGGTGCTGG-3^′^, (R) 5^′^-AGGTTCTGCGGTTTAATATCCC-3^′^; hNodal (F) 5^′^-ACCCAGCTGTGTGTACTCAA-3^′^, (R) 5^′^-TGGTAACGTTTCAGCAGAC-3^′^; hOct-4 (F) 5^′^-TATCGAGAACCGAGTGAGAG-3^′^, (R) 5^′^-TCGTTGTGCATAGTCGCT-3^′^; hPax6 (F) 5^′^-GGCGGAGTTATGTATACCTAC-3^′^, (R) 5^′^-CTTGGCCAGTATTGAGACAT-3^′^; hRex1 (F) 5^′^-AAACGGGCAAAGACAAGA-3^′^; (R) 5^′^-GCTCATAGCACACATAGCCAT-3^′^; hLefty (F) 5^′^-TGTATCCATTGAGCCCTCT-3^′^, (R) 5^′^-CAGGAAATGGAAGGACACA-3^′^; hNanog (F) 5^′^-ACCCAGCTGTGTGTACTCAA-3^′^, (R) 5^′^-GCGTCACCATTGCTATT-3^′^; hBrachyury (F) 5^′^-TGCTGCAATCCCATGACA-3^′^, (R) 5^′^-CGTTGCTCACAGACCACA-3^′^; hSOX2 (F) 5^′^-TGGGTTCGGTGGTCAAGT-3^′^, (R) 5^′^-CTCTGGTAGTGCTGGGACA-3^′^; hAFP (F) 5^′^-CTGCAAACTGACCACGCT-3^′^, (R) 5^′^-TGAGACAGCAAGCTGAGGAT-3^′^.

The PCR cycling conditions consisted of 10 min at 95°C for 1 cycle followed by 45 cycles at 95°C for 30 s, 60°C for 30 s, and 72°C for 60 s. Dissociation curve analyses confirmed that the signals corresponded to unique amplicons. Expression levels were normalized to β-actin mRNA levels for each sample obtained from parallel assays and analyzed using the LightCycler®2.0 System software package (Roche Applied Science, Indianapolis, IN, USA).

### Sphere-forming assay

ACCS cells were seeded at a density of 5 × 10^4^ cells/mL in 60-mm noncoated dishes with serum-free DMEM containing 40 ng/mL basic fibroblast growth factor (bFGF) and 20 ng/mL epidermal growth factor (EGF) for floating cultures. The cells were cultured in a humidified incubator in an atmosphere of 5% CO_2_ at 37°C, and bFGF and EGF were added to the medium every other day. After 10 days, the diameters of developed cell clusters were measured, and cell clusters with a diameter >100 μm were counted as spheres. For passaging, primary spheres (day 10) were treated with 0.05% trypsin/0.02% EDTA and dissociated into single cells, after which the cells were added to 24-well culture plates at a density of 1 × 10^4^ cells/mL in serum-free medium. The cells were cultured for a further 10 days in serum-free medium to obtain secondary spheres.

### Transfection of Brachyury and SOX2 shRNA

Cultured ACCS cells were transfected with shRNA lentiviral plasmids (pLKO.1-puro; Sigma-Aldrich) using Lipofectamine LTX (Invitrogen) according to the manufacturer’s instructions. ACCS-sh. control and ACCS-M-sh. control cells were generated by the transfection of ACCS GFP and ACCS-M GFP cells with pLKO.1-puro Control Vector (Sigma-Aldrich), respectively. ACCS-shBra and ACCS-M-shBra cells were generated by the transfection of ACCS GFP and ACCS-M GFP cells with pLKO.1-puro/sh. Brachyury (Sigma-Aldrich), respectively. Similarly, ACCS-shSOX2 and ACCS-M-shSOX2 cells were generated by the transfection of ACCS GFP and ACCS-M GFP cells with pLKO.1-puro/sh. SOX2 (Sigma-Aldrich), respectively. Colonies exhibiting resistance to puromycin (Sigma-Aldrich) were pooled from the individual transfection experiments. The expression level of Brachyury in shRNA-transfected ACCS cells was monitored by real-time RT-PCR. All transfected cells were maintained in DMEM containing 10% fetal bovine serum and 2 μg/mL puromycin (Sigma-Aldrich).

### ACCS metastatic orthotopic implantation mouse model

The animal experimental protocols were approved by the Animal Care and Use Committee of Kyushu University. Eight-week-old female athymic nude mice (BALBcAJcl-nu) were purchased from Kyudo (Fukuoka, Japan). The mice were housed in laminar flow cabinets under specific pathogen-free conditions in facilities approved by Kyushu University. For the experimental metastasis studies, 1 × 10^6^ cells in 40 μL phosphate-buffered saline (PBS) were injected into the tongue using a syringe with a 27-gauge disposable needle (TOP Plastic Syringe, Tokyo, Japan) under intraperitoneal diethyl ether anesthesia. The primary tumor volumes were measured weekly, calculated as length × width × thickness, and mice were sacrificed when the primary tumor volume reached 100 mm^3^. After sacrifice, tumors of the tongue and metastases, from tongue tumor in cervical lymph nodes, lungs, and liver were visualized macroscopically under light excitation. After visualization, the primary tumors and metastatic sites were examined pathologically and immunohistochemically.

### Immunohistochemistry

All biopsies were obtained from 21 patients who had been diagnosed with primary AdCC and treated at the Department of Oral and Maxillofacial Surgery, Kyushu University Hospital, Fukuoka, Japan, between 1993 and 2006. The protocol for this research project has been approved by a suitably constituted Ethics Committee of Kyushu University. The biopsy samples were fixed in 10% neutralized buffered formalin. Consecutive 4-μm-thick sections were cut, deparaffinized with xylene, and rehydrated in a graded alcohol series, followed by heat treatment with Target Retrieval Solution (Dako, Carpinteria, CA, USA), and then used for the histopathological and immunohistochemical analyses.

To block endogenous peroxide activity, 3% H_2_O_2_ was applied, and nonspecific reactions were blocked with 10% normal blocking serum in Tris–HCl buffer. The sections were incubated overnight at 4°C with the following primary antibodies: rabbit polyclonal anti-human Brachyury (H-210; Santa Cruz Biotechnology, Santa Cruz, CA, USA), mouse monoclonal anti-human E-cadherin (610181; BD Bioscience, California, CA, USA), and goat polyclonal anti-human vimentin (C-20; Santa Cruz Biotechnology, Santa Cruz, CA, USA). Immunostaining was performed with the Histofine SAB-PO kit (Nichirei, Tokyo, Japan), in accordance with the manufacturer’s instructions. The immunolocalization of the protein was visualized using DAB substrate kit (Nichirei). The sections were counterstained with 0.5% hematoxylin, dehydrated, cleared, and mounted. Negative control staining consisted of substituting non-immune goat serum for the primary antibodies.

### Statistical analysis

All data were displayed as mean ± SD, analyzed via analysis of variance and Student’s *t*-test, and processed by the statistical software SPSS 13.0. Statistical significance was assumed at *P* < 0.05.

## Competing interests

The authors have no potential conflicts of interest to disclose.

## Authors’ contributions

TS conceived of the study, participated in its design and coordination, performed experiments, analyzed data, performed statistical analysis, and drafted the manuscript. MS performed experiments, analyzed data, performed statistical analysis, and drafted the manuscript. II performed immunohistochemical staining. KI analyzed data and helped in drafting the manuscript. SC contributed materials and helped in drafting the manuscript. YK performed experiments, analyzed data, and performed statistical analysis. KS conceived of the study and participated in its design and coordination. All authors read and approved the final manuscript.

## Pre-publication history

The pre-publication history for this paper can be accessed here:

http://www.biomedcentral.com/1471-2407/12/377/prepub

## References

[B1] Rapidis AD Givalos N Gakiopoulou H Faratzis G Stavrianos SD Vilos GA Douzinas EE Patsouris E Adenoid cystic carcinoma of the head and neck. Clinicopathological analysis of 23 patients and review of the literature Oral Oncol 2005 41 328 335 10.1016/j.oraloncology.2004.12.004 15743696

[B2] Ampil FL Misra RP Factors influencing survival of patients with adenoid cystic carcinoma of the salivary glands J Oral Maxillofac Surg 1987 45 1005 1010 10.1016/0278-2391(87)90154-6 2826732

[B3] Ishii K Shimoda M Sugiura T Seki K Takahashi M Abe M Matsuki R Inoue Y Shirasuna K Involvement of epithelial-mesenchymal transition in adenoid cystic carcinoma metastasis Int J Oncol 2011 38 921 931 21258767 10.3892/ijo.2011.917

[B4] Tarin D Thompson EW Newgreen DF The fallacy of epithelial mesenchymal transition in neoplasia Cancer Res 2005 65 5996 6000 discussion 6000–599 10.1158/0008-5472.CAN-05-0699 16024596

[B5] Thompson EW Newgreen DF Tarin D Carcinoma invasion and metastasis: a role for epithelial-mesenchymal transition? Cancer Res 2005 65 5991 5995 discussion 5995 16024595 10.1158/0008-5472.CAN-05-0616

[B6] Grunert S Jechlinger M Beug H Diverse cellular and molecular mechanisms contribute to epithelial plasticity and metastasis Nat Rev Mol Cell Biol 2003 4 657 665 10.1038/nrm1175 12923528

[B7] Nieto MA The snail superfamily of zinc-finger transcription factors Nat Rev Mol Cell Biol 2002 3 155 166 11994736 10.1038/nrm757

[B8] Bolos V Peinado H Perez-Moreno MA Fraga MF Esteller M Cano A The transcription factor Slug represses E-cadherin expression and induces epithelial to mesenchymal transitions: a comparison with Snail and E47 repressors J Cell Sci 2003 116 499 511 10.1242/jcs.00224 12508111

[B9] Huber MA Kraut N Beug H Molecular requirements for epithelial-mesenchymal transition during tumor progression Curr Opin Cell Biol 2005 17 548 558 10.1016/j.ceb.2005.08.001 16098727

[B10] Yang J Mani SA Donaher JL Ramaswamy S Itzykson RA Come C Savagner P Gitelman I Richardson A Weinberg RA Twist, a master regulator of morphogenesis, plays an essential role in tumor metastasis Cell 2004 117 927 939 10.1016/j.cell.2004.06.006 15210113

[B11] Cano A Perez-Moreno MA Rodrigo I Locascio A Blanco MJ del Barrio MG Portillo F Nieto MA The transcription factor snail controls epithelial-mesenchymal transitions by repressing E-cadherin expression Nat Cell Biol 2000 2 76 83 10.1038/35000025 10655586

[B12] Yang MH Hsu DS Wang HW Wang HJ Lan HY Yang WH Huang CH Kao SY Tzeng CH Tai SK Bmi1 is essential in Twist1-induced epithelial-mesenchymal transition Nat Cell Biol 2010 12 982 992 10.1038/ncb2099 20818389

[B13] Kispert A Herrmann BG Leptin M Reuter R Homologs of the mouse Brachyury gene are involved in the specification of posterior terminal structures in Drosophila, Tribolium, and Locusta Genes Dev 1994 8 2137 2150 10.1101/gad.8.18.2137 7958884

[B14] Behr R Heneweer C Viebahn C Denker HW Thie M Epithelial-mesenchymal transition in colonies of rhesus monkey embryonic stem cells: a model for processes involved in gastrulation Stem Cells 2005 23 805 816 10.1634/stemcells.2004-0234 15917476

[B15] Vidricaire G Jardine K McBurney MW Expression of the Brachyury gene during mesoderm development in differentiating embryonal carcinoma cell cultures Development 1994 120 115 122 8119120 10.1242/dev.120.1.115

[B16] Fernando RI Litzinger M Trono P Hamilton DH Schlom J Palena C The T-box transcription factor Brachyury promotes epithelial-mesenchymal transition in human tumor cells J Clin Invest 2010 120 533 544 10.1172/JCI38379 20071775 PMC2810072

[B17] Reya T Morrison SJ Clarke MF Weissman IL Stem cells, cancer, and cancer stem cells Nature 2001 414 105 111 10.1038/35102167 11689955

[B18] Thiery JP Epithelial-mesenchymal transitions in development and pathologies Curr Opin Cell Biol 2003 15 740 746 10.1016/j.ceb.2003.10.006 14644200

[B19] Mani SA Guo W Liao MJ Eaton EN Ayyanan A Zhou AY Brooks M Reinhard F Zhang CC Shipitsin M The epithelial-mesenchymal transition generates cells with properties of stem cells Cell 2008 133 704 715 10.1016/j.cell.2008.03.027 18485877 PMC2728032

[B20] Berry NB Bapat SA Ovarian cancer plasticity and epigenomics in the acquisition of a stem-like phenotype J Ovarian Res 2008 1 8 10.1186/1757-2215-1-8 19025622 PMC2612659

[B21] Ahmed N Abubaker K Findlay J Quinn M Epithelial mesenchymal transition and cancer stem cell-like phenotypes facilitate chemoresistance in recurrent ovarian cancer Curr Cancer Drug Targets 2010 10 268 278 10.2174/156800910791190175 20370691

[B22] Blick T Hugo H Widodo E Waltham M Pinto C Mani SA Weinberg RA Neve RM Lenburg ME Thompson EW Epithelial mesenchymal transition traits in human breast cancer cell lines parallel the CD44(hi/)CD24 (lo/-) stem cell phenotype in human breast cancer J Mammary Gland Biol Neoplasia 2010 15 235 252 10.1007/s10911-010-9175-z 20521089

[B23] Alison MR Lim SM Nicholson LJ Cancer stem cells: problems for therapy? J Pathol 2011 223 147 161 21125672 10.1002/path.2793

[B24] Wang Z Li Y Ahmad A Azmi AS Kong D Banerjee S Sarkar FH Targeting miRNAs involved in cancer stem cell and EMT regulation: An emerging concept in overcoming drug resistance Drug Resist Updat 2010 13 109 118 10.1016/j.drup.2010.07.001 20692200 PMC2956795

[B25] Vazquez-Martin A Oliveras-Ferraros C Cufi S Del Barco S Martin-Castillo B Menendez JA Metformin regulates breast cancer stem cell ontogeny by transcriptional regulation of the epithelial-mesenchymal transition (EMT) status Cell Cycle 2010 9 3807 3814 10.4161/cc.9.18.13131 20890129

[B26] Sarkar FH Li Y Wang Z Kong D NF-kappaB signaling pathway and its therapeutic implications in human diseases Int Rev Immunol 2008 27 293 319 10.1080/08830180802276179 18853341

[B27] Raimondi C Gianni W Cortesi E Gazzaniga P Cancer stem cells and epithelial-mesenchymal transition: revisiting minimal residual disease Curr Cancer Drug Targets 2010 10 496 508 10.2174/156800910791517154 20384575

[B28] Roussos ET Keckesova Z Haley JD Epstein DM Weinberg RA Condeelis JS AACR special conference on epithelial-mesenchymal transition and cancer progression and treatment Cancer Res 2010 70 7360 7364 10.1158/0008-5472.CAN-10-1208 20823151

[B29] Herrmann BG Labeit S Poustka A King TR Lehrach H Cloning of the T gene required in mesoderm formation in the mouse Nature 1990 343 617 622 10.1038/343617a0 2154694

[B30] Kispert A Hermann BG The Brachyury gene encodes a novel DNA binding protein EMBO J 1993 12 4898 4899 8223498 10.1002/j.1460-2075.1993.tb06179.x PMC413945

[B31] Edwards YH Putt W Lekoape KM Stott D Fox M Hopkinson DA Sowden J The human homolog T of the mouse T(Brachyury) gene; gene structure, cDNA sequence, and assignment to chromosome 6q27 Genome Res 1996 6 226 233 10.1101/gr.6.3.226 8963900

[B32] Palena C Polev DE Tsang KY Fernando RI Litzinger M Krukovskaya LL Baranova AV Kozlov AP Schlom J The human T-box mesodermal transcription factor Brachyury is a candidate target for T-cell-mediated cancer immunotherapy Clin Cancer Res 2007 13 2471 2478 10.1158/1078-0432.CCR-06-2353 17438107

[B33] Creighton CJ Chang JC Rosen JM Epithelial-mesenchymal transition (EMT) in tumor-initiating cells and its clinical implications in breast cancer J Mammary Gland Biol Neoplasia 2010 15 253 260 10.1007/s10911-010-9173-1 20354771

[B34] Ding W You H Dang H LeBlanc F Galicia V Lu SC Stiles B Rountree CB Epithelial-to-mesenchymal transition of murine liver tumor cells promotes invasion Hepatology 2010 52 945 953 10.1002/hep.23748 20564331 PMC3032356

[B35] Sarkar FH Li Y Wang Z Kong D Pancreatic cancer stem cells and EMT in drug resistance and metastasis Minerva Chir 2009 64 489 500 19859039 PMC2878773

[B36] Monteiro J Fodde R Cancer stemness and metastasis: therapeutic consequences and perspectives Eur J Cancer 2010 46 1198 1203 10.1016/j.ejca.2010.02.030 20303259

[B37] Singh A Settleman J EMT, cancer stem cells and drug resistance: an emerging axis of evil in the war on cancer Oncogene 2010 29 4741 4751 10.1038/onc.2010.215 20531305 PMC3176718

[B38] DiMeo TA Anderson K Phadke P Fan C Perou CM Naber S Kuperwasser C A novel lung metastasis signature links Wnt signaling with cancer cell self-renewal and epithelial-mesenchymal transition in basal-like breast cancer Cancer Res 2009 69 5364 5373 10.1158/0008-5472.CAN-08-4135 19549913 PMC2782448

[B39] Aktas B Tewes M Fehm T Hauch S Kimmig R Kasimir-Bauer S Stem cell and epithelial-mesenchymal transition markers are frequently overexpressed in circulating tumor cells of metastatic breast cancer patients Breast Cancer Res 2009 11 R46 10.1186/bcr2333 19589136 PMC2750105

[B40] Kong D Banerjee S Ahmad A Li Y Wang Z Sethi S Sarkar FH Epithelial to mesenchymal transition is mechanistically linked with stem cell signatures in prostate cancer cells PLoS One 2010 5 e12445 10.1371/journal.pone.0012445 20805998 PMC2929211

[B41] Wendt MK Allington TM Schiemann WP Mechanisms of the epithelial-mesenchymal transition by TGF-beta Future Oncol 2009 5 1145 1168 10.2217/fon.09.90 19852727 PMC2858056

[B42] Kabashima A Higuchi H Takaishi H Matsuzaki Y Suzuki S Izumiya M Iizuka H Sakai G Hozawa S Azuma T Hibi T Side population of pancreatic cancer cells predominates in TGF-beta-mediated epithelial to mesenchymal transition and invasion Int J Cancer 2009 124 2771 2779 10.1002/ijc.24349 19296540

[B43] Bhat-Nakshatri P Appaiah H Ballas C Pick-Franke P Goulet R Jr Badve S Srour EF Nakshatri H SLUG/SNAI2 and tumor necrosis factor generate breast cells with CD44+/CD24- phenotype BMC Cancer 2010 10 411 10.1186/1471-2407-10-411 20691079 PMC3087321

[B44] Kalluri R EMT: when epithelial cells decide to become mesenchymal-like cells J Clin Invest 2009 119 1417 1419 10.1172/JCI39675 19487817 PMC2689122

[B45] Zeisberg M Neilson EG Biomarkers for epithelial-mesenchymal transitions J Clin Invest 2009 119 1429 1437 10.1172/JCI36183 19487819 PMC2689132

[B46] Thiery JP Sleeman JP Complex networks orchestrate epithelial-mesenchymal transitions Nat Rev Mol Cell Biol 2006 7 131 142 10.1038/nrm1835 16493418

[B47] Peinado H Olmeda D Cano A Snail, Zeb and bHLH factors in tumour progression: an alliance against the epithelial phenotype? Nat Rev Cancer 2007 7 415 428 10.1038/nrc2131 17508028

[B48] Dumont N Wilson MB Crawford YG Reynolds PA Sigaroudinia M Tlsty TD Sustained induction of epithelial to mesenchymal transition activates DNA methylation of genes silenced in basal-like breast cancers Proc Natl Acad Sci USA 2008 105 14867 14872 10.1073/pnas.0807146105 18806226 PMC2567459

[B49] Kalluri R Weinberg RA The basics of epithelial-mesenchymal transition J Clin Invest 2009 119 1420 1428 10.1172/JCI39104 19487818 PMC2689101

[B50] Acloque H Adams MS Fishwick K Bronner-Fraser M Nieto MA Epithelial-mesenchymal transitions: the importance of changing cell state in development and disease J Clin Invest 2009 119 1438 1449 10.1172/JCI38019 19487820 PMC2689100

[B51] Fuxe J Vincent T Garcia de Herreros A Transcriptional crosstalk between TGFbeta and stem cell pathways in tumor cell invasion: Role of EMT promoting Smad complexes Cell Cycle 2010 9 2363 2374 10.4161/cc.9.12.12050 20519943

[B52] Lindley LE Briegel KJ Molecular characterization of TGFbeta-induced epithelial-mesenchymal transition in normal finite lifespan human mammary epithelial cells Biochem Biophys Res Commun 2010 399 659 664 10.1016/j.bbrc.2010.07.138 20691661

[B53] Taube JH Herschkowitz JI Komurov K Zhou AY Gupta S Yang J Hartwell K Onder TT Gupta PB Evans KW Core epithelial-to-mesenchymal transition interactome gene-expression signature is associated with claudin-low and metaplastic breast cancer subtypes Proc Natl Acad Sci USA 2010 107 15449 15454 10.1073/pnas.1004900107 20713713 PMC2932589

[B54] Muller CW Herrmann BG Crystallographic structure of the T domain-DNA complex of the Brachyury transcription factor Nature 1997 389 884 888 10.1038/39929 9349824

[B55] Rodriguez M Aladowicz E Lanfrancone L Goding CR Tbx3 represses E-cadherin expression and enhances melanoma invasiveness Cancer Res 2008 68 7872 7881 10.1158/0008-5472.CAN-08-0301 18829543

[B56] Savagner P Kusewitt DF Carver EA Magnino F Choi C Gridley T Hudson LG Developmental transcription factor slug is required for effective re-epithelialization by adult keratinocytes J Cell Physiol 2005 202 858 866 10.1002/jcp.20188 15389643

[B57] Savagner P Yamada KM Thiery JP The zinc-finger protein slug causes desmosome dissociation, an initial and necessary step for growth factor-induced epithelial-mesenchymal transition J Cell Biol 1997 137 1403 1419 10.1083/jcb.137.6.1403 9182671 PMC2132541

[B58] Hotta K Takahashi H Satoh N Gojobori T Brachyury-downstream gene sets in a chordate, Ciona intestinalis: integrating notochord specification, morphogenesis and chordate evolution Evol Dev 2008 10 37 51 10.1111/j.1525-142X.2007.00212.x 18184356

